# Supplementary figures and images for: Early transcription factor activation distinguishes symbiotic from non-symbiotic bacteria during microbiome processing in a sponge
Source: ISME J. 2026 Jun 11;20(1):wrag150. doi: 10.1093/ismejo/wrag150 (PMC13372038; doi:10.1093/ismejo/wrag150)

Supplementary Fig. S10

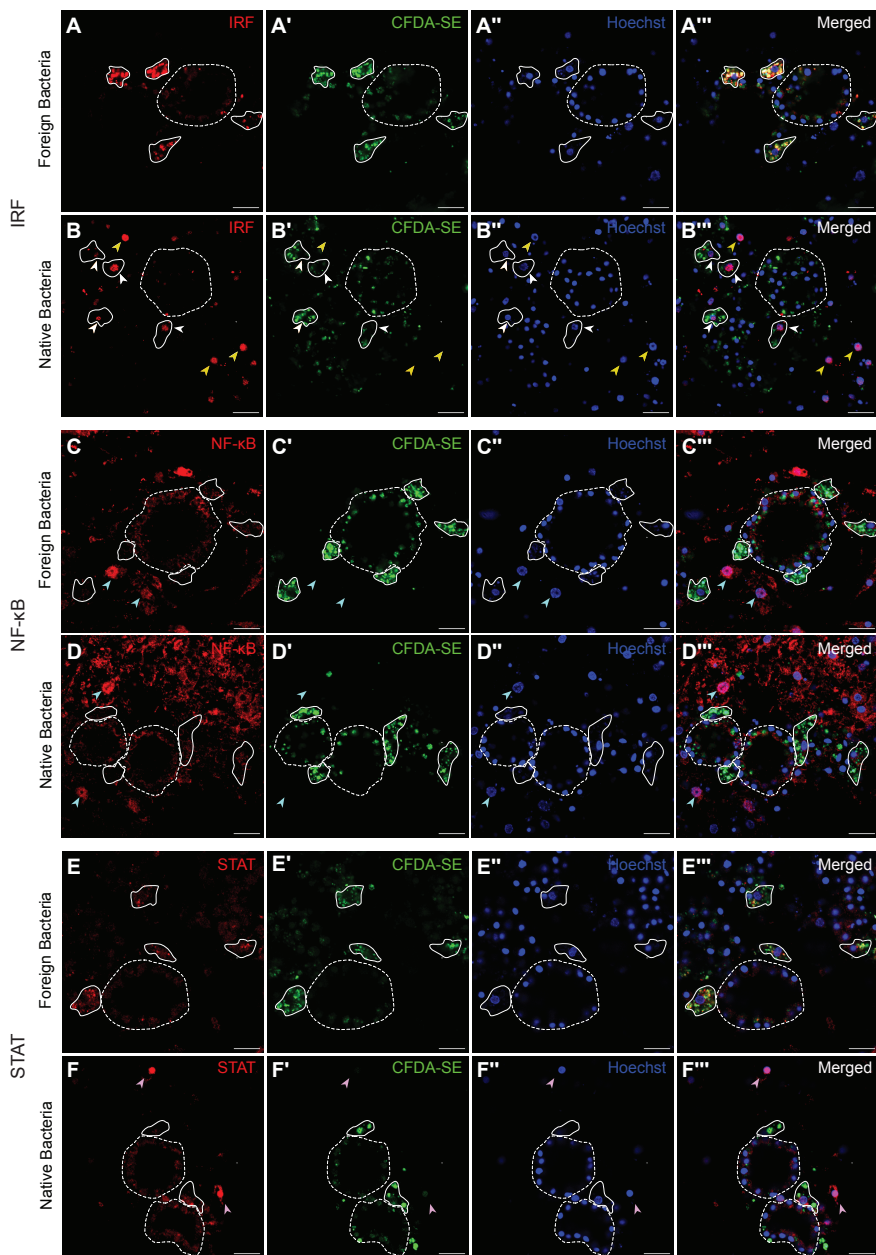

Supplement: Supplementary_material_wrag150 [file supplementary_material_wrag150.zip › Fig._S10_wrag150.pdf]

Supplementary Fig. S11

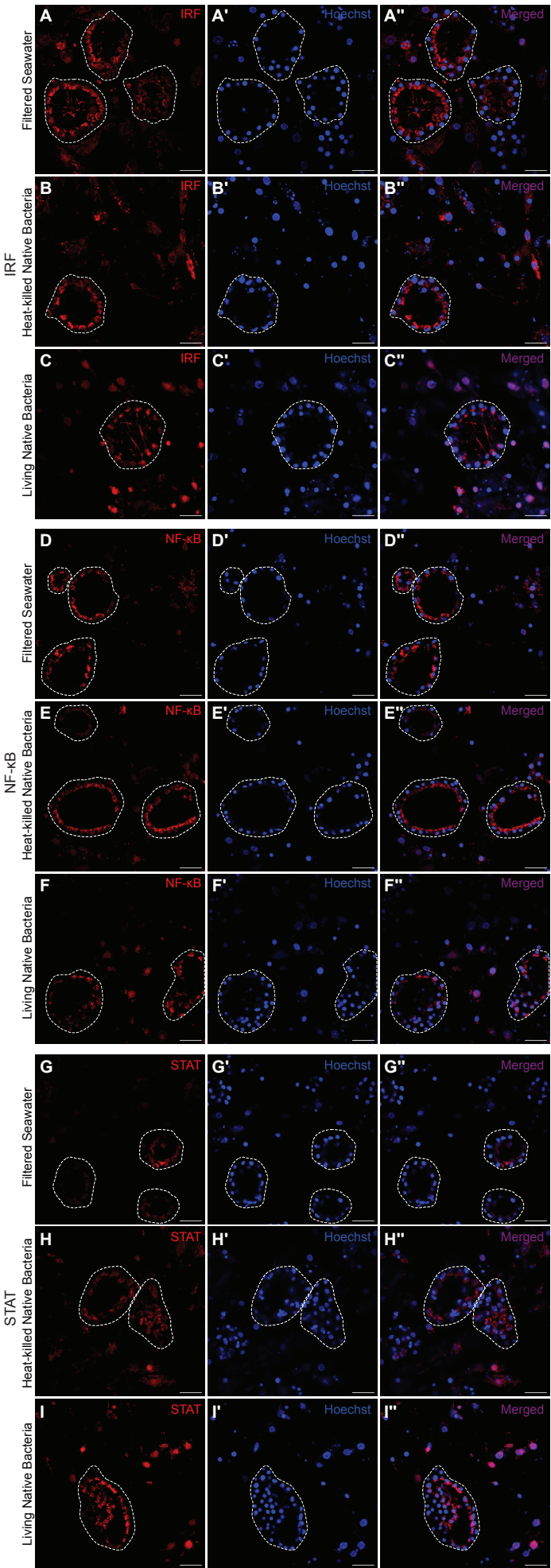

Supplement: Supplementary_material_wrag150 [file supplementary_material_wrag150.zip › Fig._S11_wrag150.pdf]

# Supplementary Fig. S1

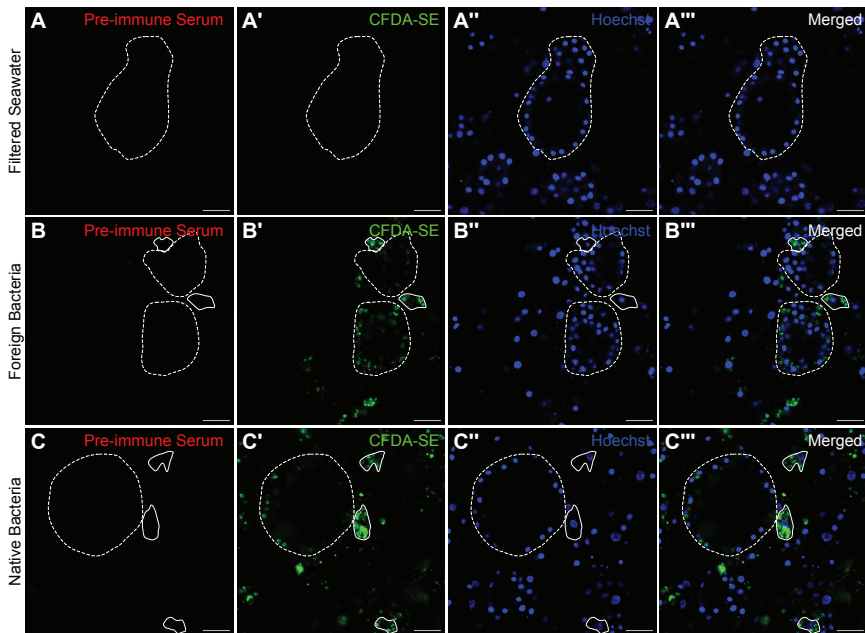

Supplement: Supplementary_material_wrag150 [file supplementary_material_wrag150.zip › Fig._S1_wrag150.pdf]

Supplementary Fig. S2

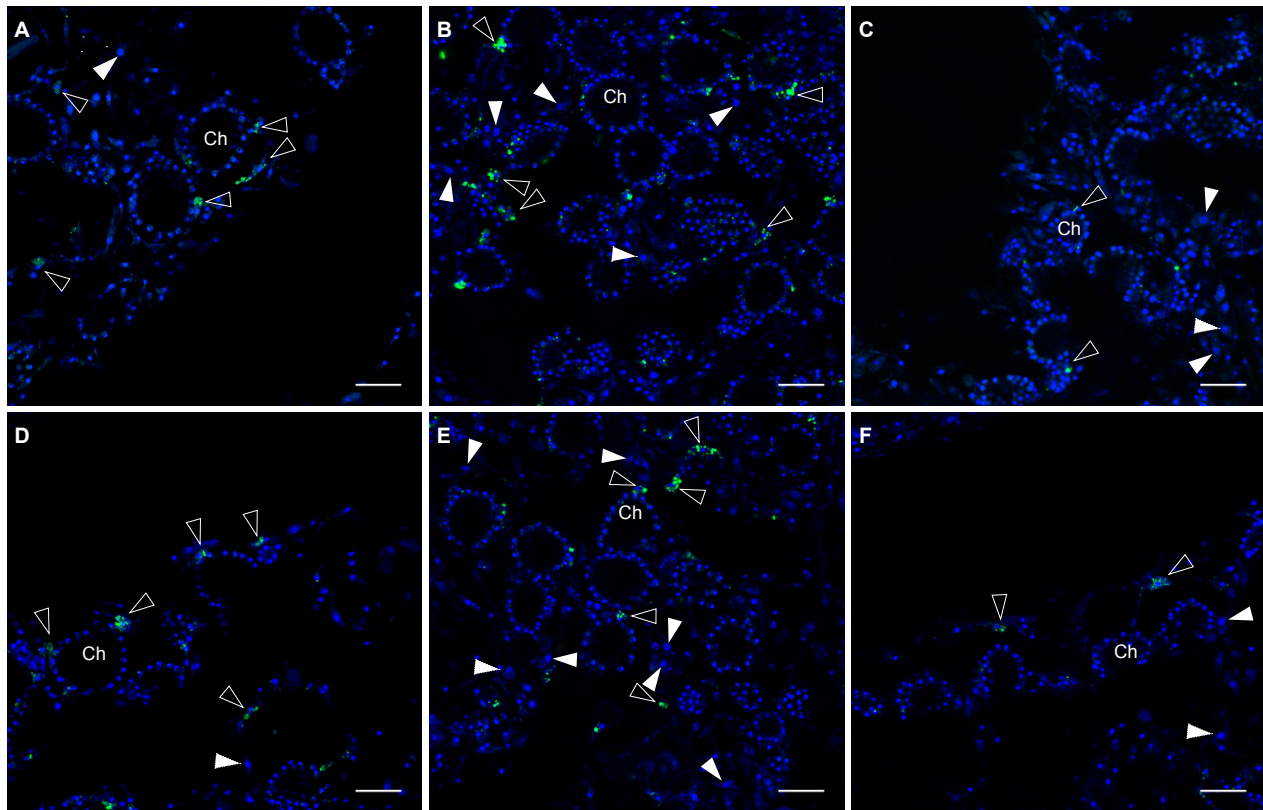

Supplement: Supplementary_material_wrag150 [file supplementary_material_wrag150.zip › Fig._S2_wrag150.pdf]

Supplementary Fig. S3

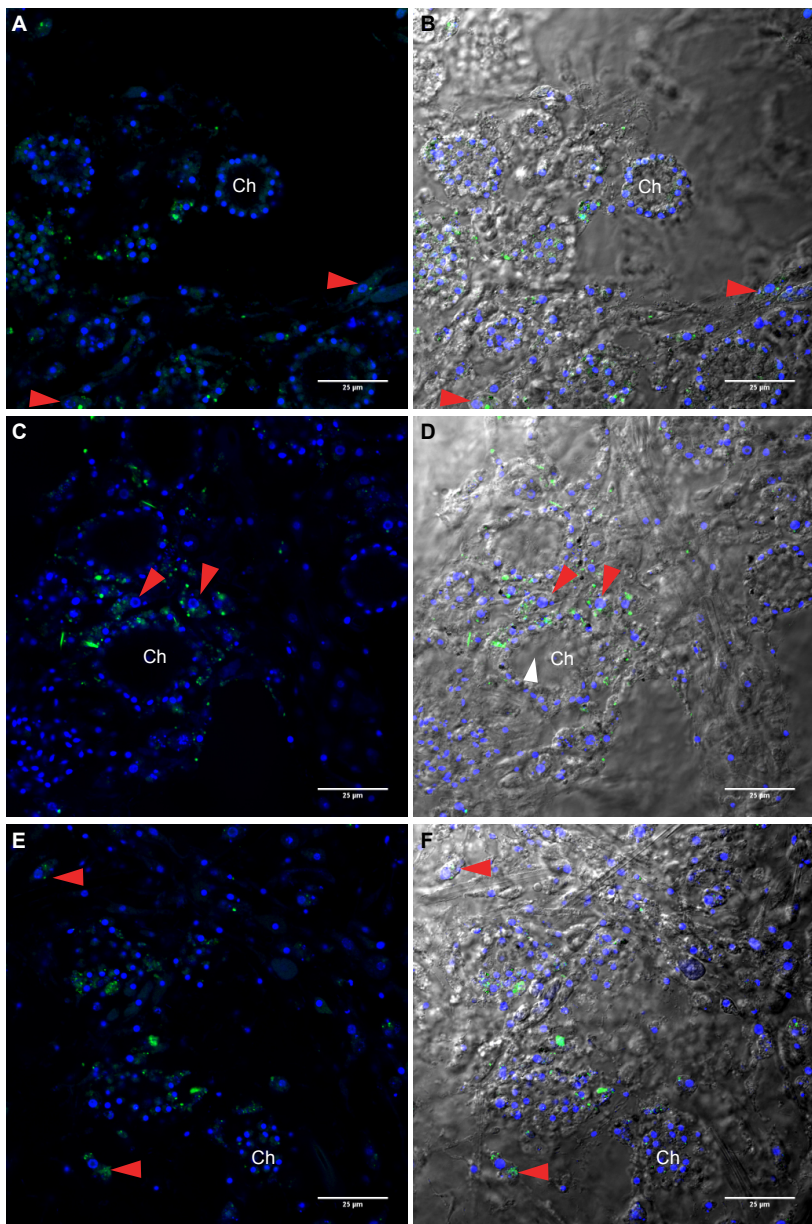

Supplement: Supplementary_material_wrag150 [file supplementary_material_wrag150.zip › Fig._S3_wrag150.pdf]

## Supplementary Fig. S4

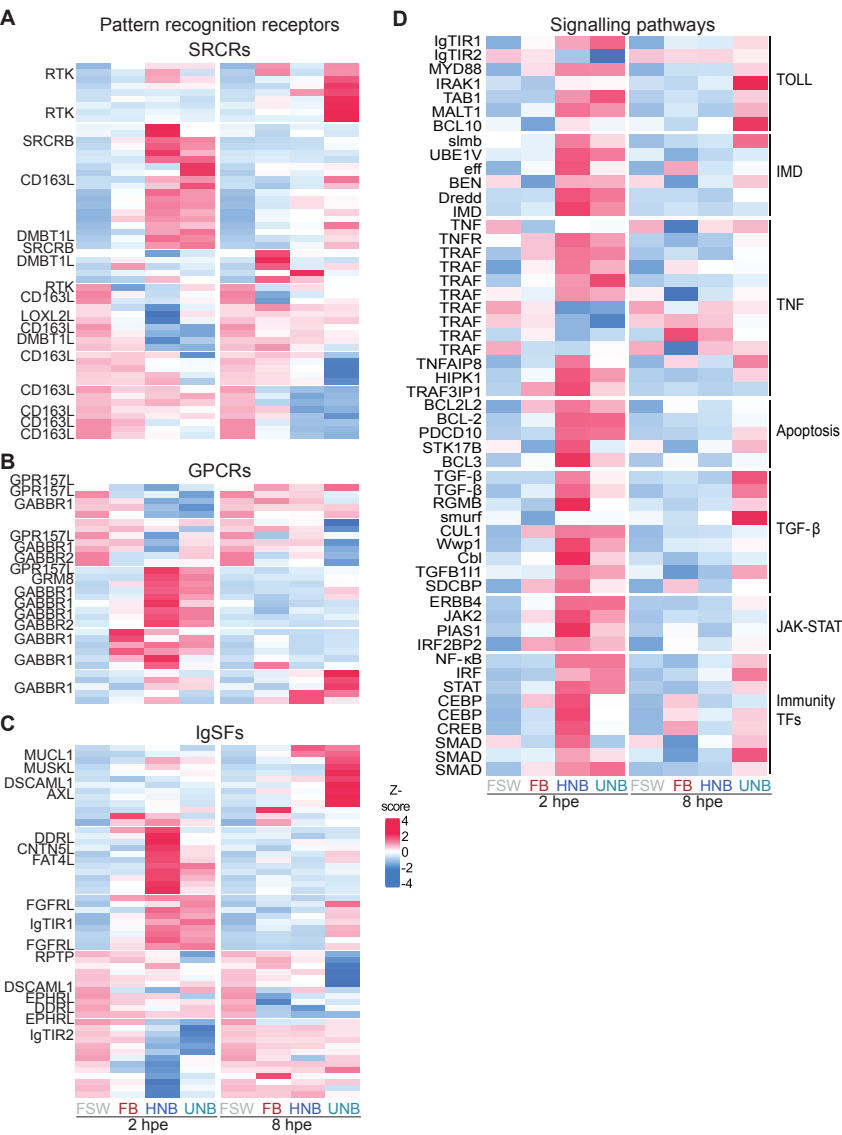

Supplement: Supplementary_material_wrag150 [file supplementary_material_wrag150.zip › Fig._S4_wrag150.pdf]

Supplementary Fig. S5

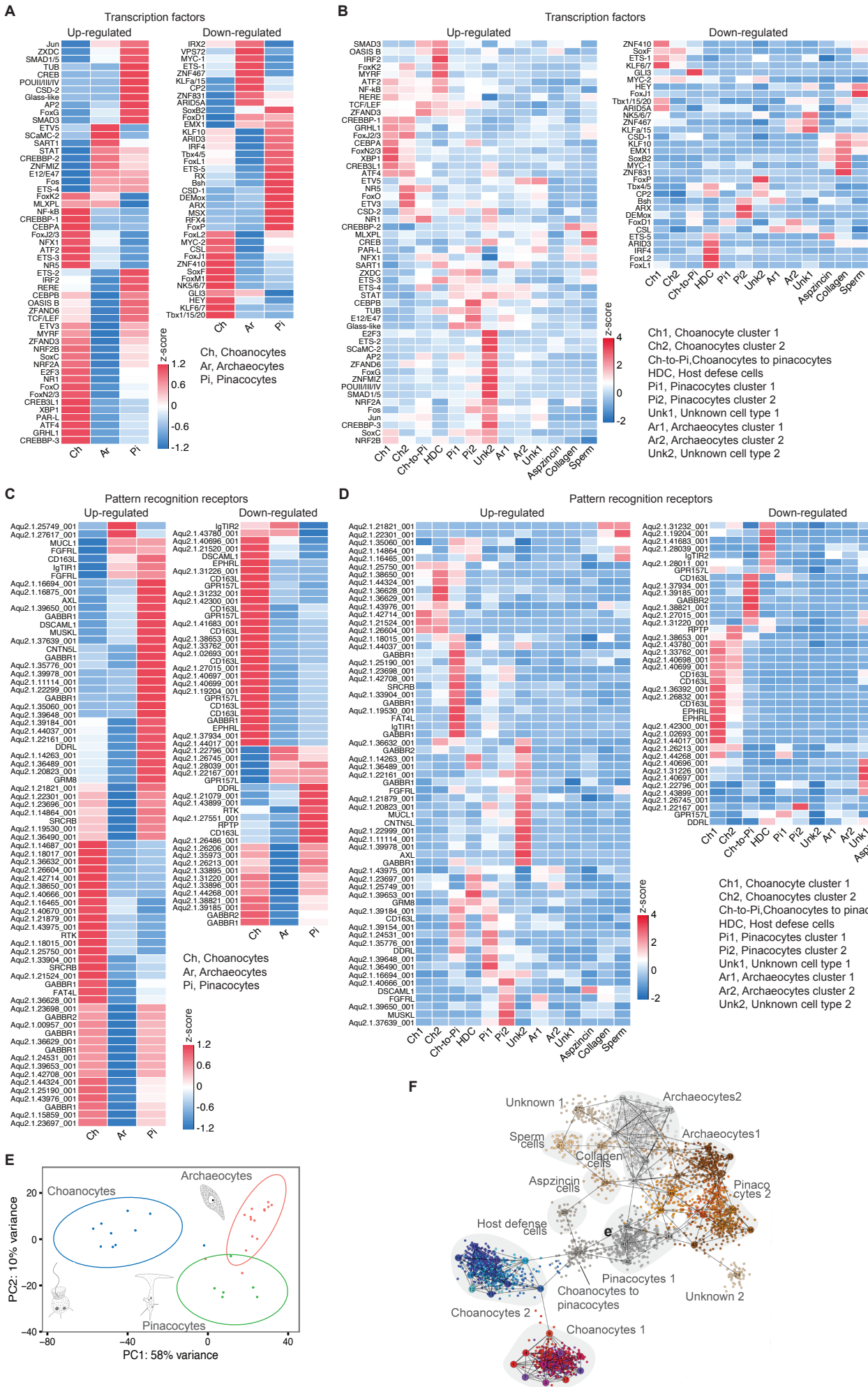

Supplement: Supplementary_material_wrag150 [file supplementary_material_wrag150.zip › Fig._S5_wrag150.pdf]

# Supplementary Fig. S6

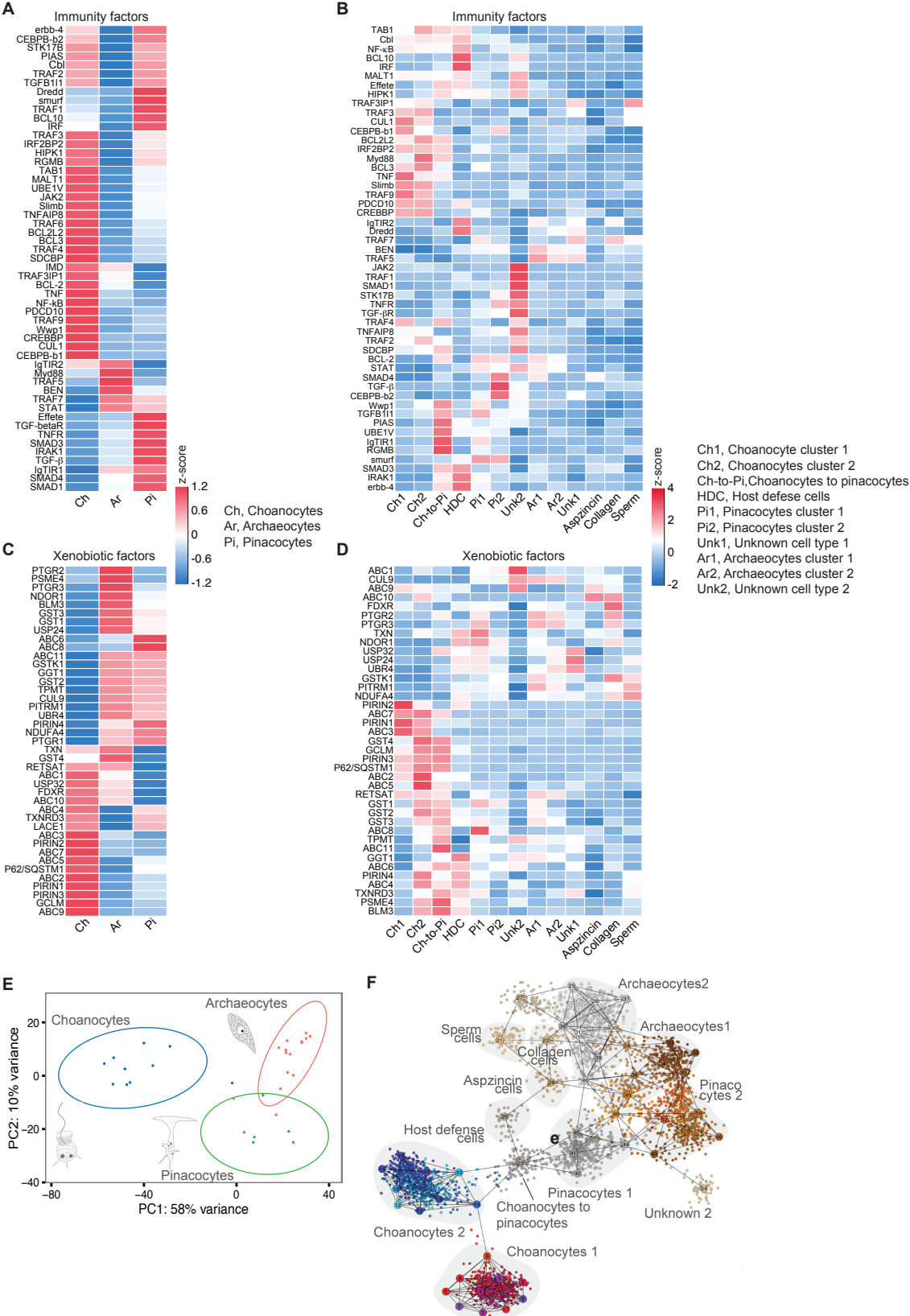

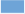

Supplement: Supplementary_material_wrag150 [file supplementary_material_wrag150.zip › Fig._S6_wrag150.pdf]

Supplementary Fig. S7

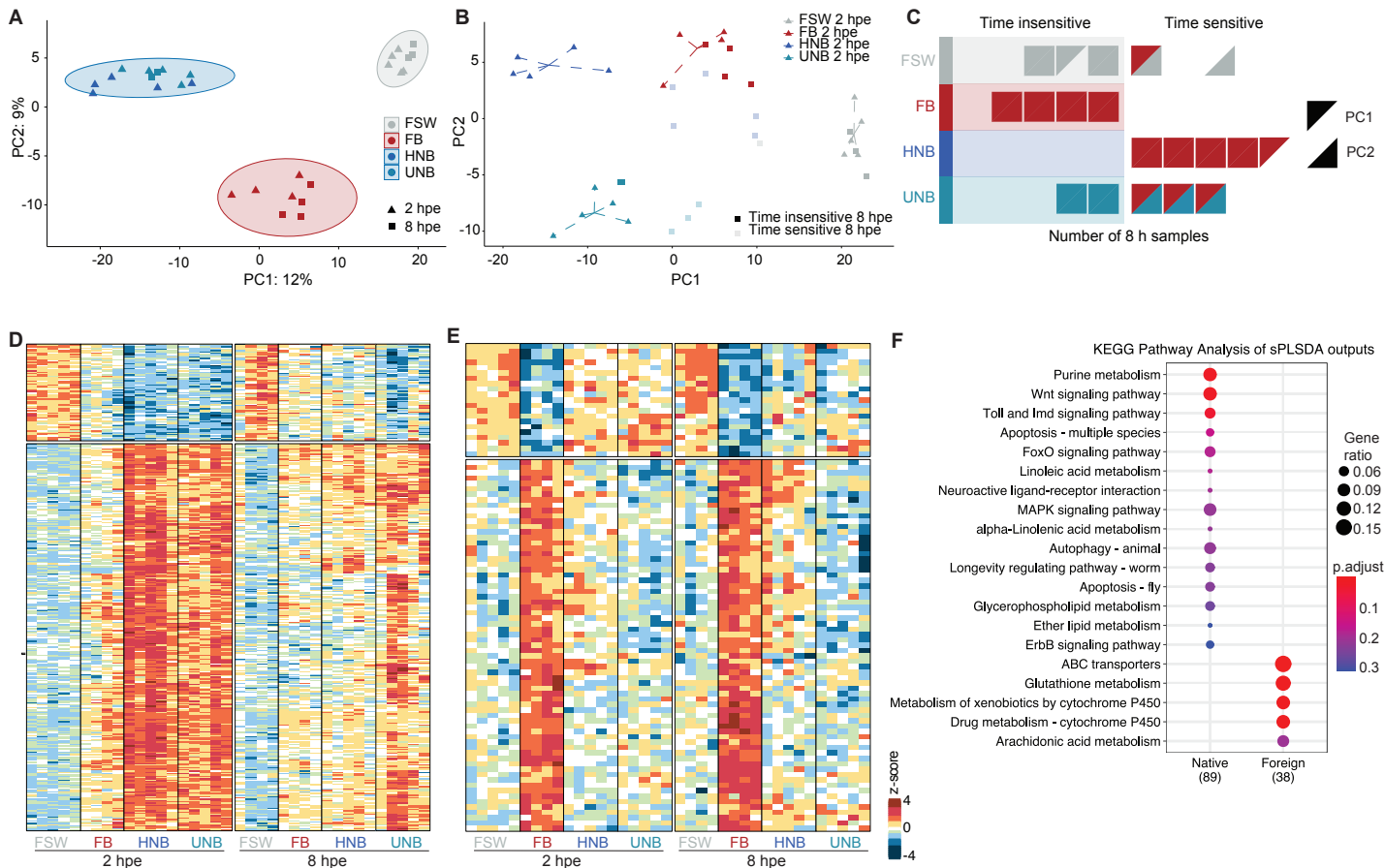

Supplement: Supplementary_material_wrag150 [file supplementary_material_wrag150.zip › Fig._S7_wrag150.pdf]

Supplementary Fig. S8

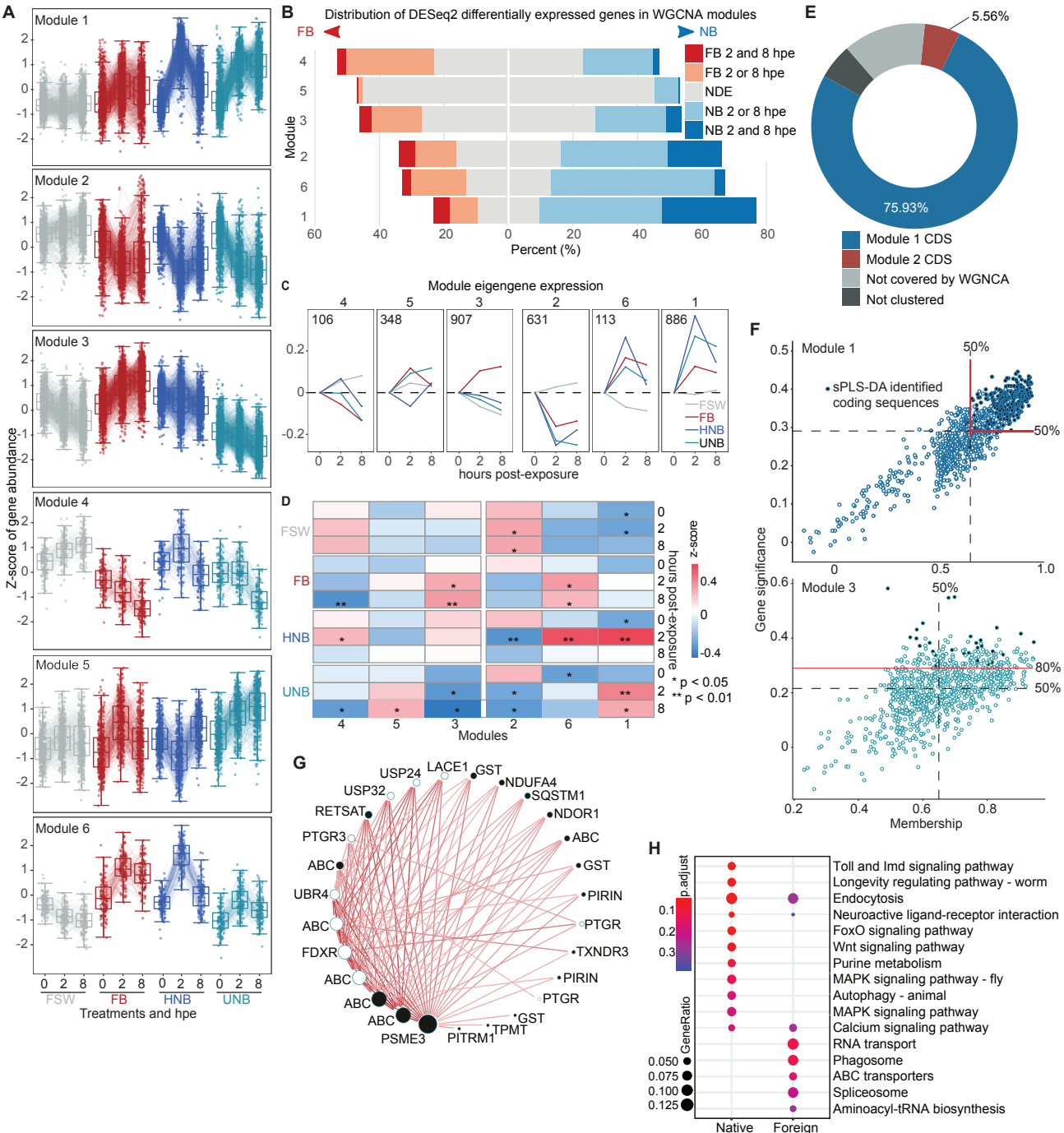

Supplement: Supplementary_material_wrag150 [file supplementary_material_wrag150.zip › Fig._S8_wrag150.pdf]

Supplementary Fig. S9

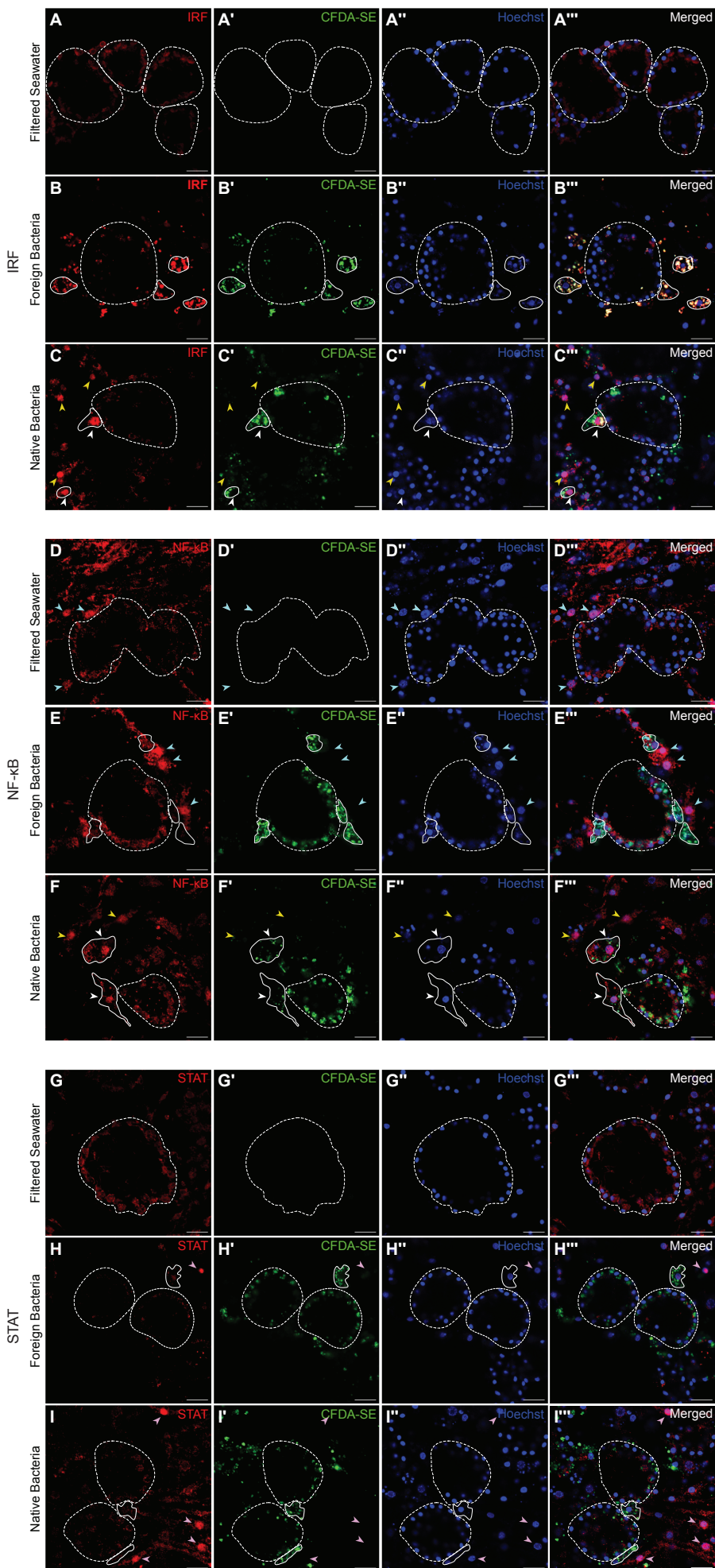

Supplement: Supplementary_material_wrag150 [file supplementary_material_wrag150.zip › Fig._S9_wrag150.pdf]
